# Behavioral and Synaptic Circuit Features in a Zebrafish Model of Fragile X Syndrome

**DOI:** 10.1371/journal.pone.0051456

**Published:** 2013-03-11

**Authors:** Ming-Chong Ng, Yi-Ling Yang, Kwok-Tung Lu

**Affiliations:** 1 Department of Life Science, National Taiwan Normal University, Taipei, Taiwan; 2 Department of Biochemical Science and Technology, National Chia-Yi University, Chia-Yi, Taiwan; University of Medicine & Dentistry of NJ - New Jersey Medical School, United States of America

## Abstract

Fragile X syndrome (FXS) is the most frequent inherited form of human mental retardation. It is characterized by cognitive impairment and physical and behavioral problems and is caused by the silencing of *fmr1* transcription and the absence of the *fmr1* protein (FMRP). Recently, animal models of FXS have greatly facilitated the investigation of the molecular and cellular mechanisms of this loss-of-function disorder. The present study was aimed to further characterize the role of FMRP in behavior and synaptic function by using *fmr1* knockout zebrafish. In adult zebrafish, we found that *fmr1* knockout produces the anxiolytic-like responses of increased exploratory behavior in light/dark and open-field tests and avoidance learning impairment. Furthermore, electrophysiological recordings from telencephalic slice preparations of knockout fish displayed markedly reduced long-term potentiation and enhanced long-term depression compared to wild-type fish; however, basal glutamatergic transmission and presynaptic function at the lateral (Dl) and medial (Dm) division of the dorsal telencephalon synapse remained normal. Taken together, our study not only evaluates the mechanism of FRMP but also suggests that zebrafish have valuable potential as a complementary vertebrate model in studying the molecular pathogenesis of human fragile X syndrome.

## Introduction

The onset rate of fragile X syndrome (FXS) is approximately one in 4000 males and one in 8000 females affected [1], [2]. The clinical features of FXS include attention deficits, hyperactivity, social deficits, anxiety disorder and deficits in cognitive flexibility [3]. This syndrome is most commonly caused by a triplet repeat expansion (CGG) mutation in the *fmr1* gene that encodes FMRP, the fragile X mental retardation protein [4], [5], [6]. When the CGG expansions within the 5′ untranslated region (UTR) of the *fmr1* gene exceed 200 repeats (the full mutation), the hypermethylation [7] and deacetylation [8] of *fmr1* result, leading to the silencing of *fmr1* transcription and the absence of the FMR1 protein (FMRP).

FMRP, a cytoplasmic mRNA-binding protein, is widely expressed in various tissues with the most abundant expression in the brain and testes of mammalians [9], [10]. For example, FMRP is expressed in neurons, particularly those of the hippocampus, amygdala, and in the Purkinje cells of the cerebellum [10], [11]. In addition, the functional domains of FMRP that are evolutionarily conserved between humans and Drosophila include the nuclear localization signal (NLS) and two KH domains, the nuclear export signal (NES) and an RGG box. In humans, FMRP is highly expressed in neurons of the brain, where it is suggested to play an important role in the regulation of local mRNA translation within neuronal dendritic spines and altered synaptic function [12].

Animal models of FXS have greatly facilitated the investigation of the molecular and cellular mechanism of this disorder. For example, *fmr1* knockout (KO) mice were characterized as having several behavioral profiles similar to those of fragile X patients, including hyperactivity, reduced anxiety-related behavior, and learning and memory deficits [13], [14]. Synapse function is closely correlated with dendritic spine morphology and synaptic activity. Indeed, abnormalities of the dendritic spine is a significant neuroanatomical defect in FXS patients [15] and *fmr1* KO mice [16]. Therefore, the findings of a spine morphological phenotype indicate a possible defect in synaptic plasticity in FXS that could result in the cognitive disability phenotype. Long-term potentiation (LTP) and long-term depression (LTD) are the key cellular mechanisms underlying learning and memory processes [17], [18]. Consistent with behavioral learning deficits, electrophysiological studies have reported the loss of LTP in the anterior cingulate cortex (ACC) and the lateral amygdala of *fmr1* KO mice [19]. However, previous studies have also shown that a lack of FMRP may lead to exaggerated metabotropic glutamate receptor (mGluR) signaling and enhanced hippocampal LTD in *fmr1* KO mice [20].

The zebrafish is a small tropical freshwater teleost native to South-East Asia. Zebrafish were first used for biological research purposes by Dr. George Streisinger, in 1981 [21], [22], [23]. As a relatively simple vertebrate species, the zebrafish is a popular model organism for developmental and genetic studies [24]. It is also an excellent model for biomedical research, and the advantages of this model include genomic and physiological homology with humans, external fertilization, and large numbers of fertilized eggs. Most importantly, the transparency of the zebrafish embryo allows one to visualize the processes of embryogenesis and morphogenesis in early developmental stages. Moreover, the small size of zebrafish enables cost-efficient maintenance of numerous adult individuals. It is also possible to obtain and maintain the transgenic strains of zebrafish at a low cost.

Rupp and colleagues demonstrated that the basic components of the adult zebrafish brain are very similar to those of land vertebrates [25]. Due to the accumulated genetic knowledge and tools developed for the zebrafish, many reports make use of the zebrafish to study complex behaviors such as seizures [26], addiction [27], and learning and memory [28], [29], [30], [31]. Therefore, zebrafish is a valuable model for studying the pathways and mechanisms of human neurological disorders and clinical treatments.

The adult zebrafish FMRP shares 72% amino acid identity with that of humans, and similar to humans, it is highly expressed the brain, including in the telencephalon, diencephalon, metencephalon, spinal cord, and cerebellum [32]. In 2009, den Broader and colleagues generated the *fmr1* knockout (KO) zebrafish, which provided a new genetic model system to study FXS [33]. However, research analyzing the phenotypic characteristics of *fmr1* KO zebrafish in adulthood is sparse. In a previous study, we reported that the telencephalon is physiologically involved in the process of fear memory formation in the inhibitory avoidance task in zebrafish [34]. The present study aimed to further characterize the effects of the loss of FMRP on cognitive phenotypes by investigating possible differences in cognitive behavior in inhibitory avoidance and synaptic plasticity at the Dl-Dm synapse of telencephalon in adult *fmr1* KO zebrafish.

## Materials and Methods

### *fmr1* knockout (KO) zebrafish

Zebrafish mutants carrying the *fmr1* ^hu2787^ allele were obtained from the Wellcome Trust Sanger Institute Zebrafish Mutant Resource. This allele carries a C432T change, which causes a premature termination at codon position 113 [33]. Fish (4–8 months of age) of both sexes were used for these experiments; *fmr1* KO and control fish of the TL background were maintained according to standard procedures [35] and following guidelines approved by the Institutional Animal Care and Use Committee (IACUC) of National Taiwan Normal University (Approval number: 10030).

### Polymerase Chain Reaction (PCR)

Adult fishes were genotyped by PCR using tissue and cell genomic DNA purification kit (Genemark, Taipei, Taiwan) according to manufacturer protocol. DNA extracted from the tails. Zebrafish genomic DNAs were prepared using Tissue and Cell Genomic DNA Purification Kit (Genemark, Taipei, Taiwan) according to manufacturer protocol. Optic density (O.D.) value of each sample was measured by a Nano spectrophotometer (NanoDrop Technologies, Inc. Wilmington, DL) at 260 and 280 nm to determine the purity and concentration of DNA. Genotyping of the present study is based on dCAPS assay (derived Cleaved Amplified Polymorphic Sequence). In this assay, a mismatch in PCR primer is used to create restriction endonuclease (RE)-sensitive polymorphism based on the target mutation. To genotype the hu2787 allele, a mismatch has been introduced into the forward primer. During PCR, this mismatch creates an RsaI restriction enzyme site in the amplified product derived from the WT DNA template. The RsaI site is not present in the PCR product containing the hu2787 mutation. A 222-bp PCR product was generated using forward primer (5′-CTA AAT GAA ATC GTC ACA TTA GAG AGG GTA) and reverse primer (5′- TCCATG ACA TCC TGC ATT AG). The amplification reaction mixture (50 µL) contained 200 ng genomic DNA, 0.5 mM of each dNTP, 1 µM of each primer, 1 unit Prozyme DNA polymerase (Protech Enterprise, Taipei, Taiwan) and 1× PCR buffer. The PCR reaction conditions began with a denaturation at 94°C for 4 min, followed by 40 cycles of 94°C for 30 seconds, 60°C for 30 seconds and 72°C for 20 seconds; lastly, 5 min at 72°C. After amplification, the PCR product was digested by RsaI restriction enzyme in 1 X restriction enzyme buffer. Finally, digested PCR products were separated by electrophoresis in 3% agarose gel. The PCR products derived from the WT template were cleaved to 193-and 29-bp DNA fragments.

### Western blot analysis

After the animals were scarified, the telencephalon brain region was quickly removed from the skull and homogenized using a T-PER tissue protein extraction reagent kit (Pierce Biotechnology, Inc., Rockford, IL) with the addition of the Halt Protease Inhibitor Cocktail. The protein concentration was determined by the Bradford protein assay, and an equal amount of protein (25 µg per sample) was subjected to SDS–10% PAGE. The proteins separated on the gel of the SDS–PAGE were transferred to a PVDF membrane (Millipore, Bedford, MA). For the immune-detection, the membrane was first blocked with 5% skim milk and 0.05% Tween in PBS for 1 h at room temperature. The primary antibodies used for the detection were rabbit anti-FMRP (1∶4,000; Gift from Dr. Willemsen, #758) antibodies. The membranes were incubated with primary antibodies overnight at 4°C and, subsequently, with HRP-conjugated secondary antibodies for 1 h at room temperature. Finally, the detected signals were visualized with enhanced chemiluminescence (Bioman Scientific Co. Ltd., Taiwan) and quantitatively analyzed by a LAS3000 digital imaging system (Fujifilm, Tokyo, Japan).

### Behavioral procedures

Prior to the initiation of behavioral protocols, zebrafish were separated into individual tanks a few days before the experiment. To elucidate the behavioral effects of FMRP deficiency, fish (10 WT and 12 KO) were initially assessed with a light/dark test, then tested 48 h later in an inhibitory avoidance test, and finally examined in an open-field test.

### Light/dark test

To examine anxiety-related behaviors, fish were tested in a light/dark test conducted in a rectangular acryl tank (7 cm height×18 cm Length×9 cm width) divided into two equally sized compartments that were demarcated by black and white coloration. The tank water level was maintained at 3 cm. Before testing, the subjects were carefully placed in the white compartment. Then, the fish were allowed to swim freely between the two compartments without a sliding door for 5 min. Behavior was recorded using a Logitech S5500 camera and Logitech Quick Capture software. The proportion of the trial that the animal spent in the white compartment was recorded. Reduced exploration of the white compartment in this test reflects high anxiety states.

### Inhibitory avoidance task (IA)

Fish were tested for emotional learning in an inhibitory avoidance task as described previously [34]. Briefly, fish underwent acclimation, one-trial training and one test session 24 h later. On day one, the fish received an acclimation trial that consisted of placing the fish in the shallow chamber for 5 min; the white, opaque guillotine door was then removed, and the fish was allowed to swim freely between the two compartments for another 5 min. In the training session, the fish was placed in the shallow compartment. After 1 min, the guillotine door to the deep compartment was opened. Once the fish entered the deep compartment, the guillotine door was closed, and a mild electric shock was applied to the deep compartment for 5 s. The fish was immediately removed from the apparatus and returned to its home tank. Twenty-four hours later, latency to enter the deep compartment of the apparatus was recorded to a maximum of 300 sec.

### Open-field test

At the end of the retention test, the animals were placed into a transparent cylindrical tank (20 cm in height and 22 cm in diameter) for 10 min to test their spontaneous motor activity. The water level was maintained at 4 cm. Behavior was detected using an EthoVision video tracking system (Noldus Information Technology, Leesburg, VA, U S A). The total distance swam and the mean speed was measured for statistical analyses.

### Extracellular field potential recordings

Acute telencephalic slice preparation was similar to that described previously [36]. Briefly, fish were euthanized by exposing them to an ice-cold (0∼4°C), artificially oxygenated cerebrospinal fluid (aCSF) solution consisting of the following: 117 mM NaCl, 4.7 mM KCl, 1.2 mM NaH_2_PO_4_, 2.5 mM NaHCO_3_, 1.2 mM MgCl_2_, 2.5 mM CaCl_2_, and 11 mM d-(+)-glucose. Their brains were quickly removed under the aCSF solution. Transverse telencephalic slices (300 µm) were prepared using a vibrotome (MA752, Campden Instruments Ltd., UK) in ice-cold aCSF. Slices were then incubated in the aCSF solution, which was bubbled continuously with 95% O_2_/5% CO_2_ for at least 1 h prior to recordings at room temperature.

Extracellular population spikes (PSs) were recorded using a 64-channel multi-electrode dish (MED64) system (Alpha MED Sciences, Tokyo, Japan) with a sample rate of 20 kHz. Recordings were performed with an 8×8 array of planar microelectrodes. Each electrode was 20×20 µm in size, and the inter-electrode spacing was 100 µm. Telencephalic slices were placed in a recording chamber and perfused with aCSF (30°C) at a flow rate of 1–2 ml/min via a peristaltic pump (Gilson Minupuls 3, Villiers Le Bel, France).A nylon mesh and a stainless steel wire were used to secure slice position and contact with electrodes during perfusion. Stimulus intensity was adjusted to evoke 40–50% of the maximal stimulation response. Test stimuli were 0.2 ms pulses every 20 s, and responses were recorded for 15 min prior to beginning the experimental treatments to assure stability of responses. Every three consecutive responses were pooled and averaged for data analysis.

Basal synaptic transmission was measured by plotting the current applied to the stimulating electrode (40–150 µA) against the amplitude of population spike responses to generate input–output curves (I/O curves). Paired-pulse facilitation was assessed by applying pairs of stimuli at varying inter-pulse intervals (20, 50, 100, 150, and 200 ms). The paired pulse ratio (PPR) was determined by calculating the ratio of the average amplitude of the second response to the first. Each trace corresponds to an average over 3 trials. After stable baseline recording, LTP was elicited by HFS protocols consisting of three stimulus trains of 100 pulses (at 100 Hz) with 20 s inter-train intervals. LTD was induced by low frequency stimulation that consisted of 1 Hz stimulation for 20 min. The magnitudes of both LTP and LTD were measured, post-induction, as an average of 10 min at the end of the recording period.

### Statistical analysis

Statistical analysis was performed using SPSS version 12.0 (SPSS, Chicago). In electrophysiological experiments, each trace is the average of three consecutive responses. LTP plots were normalized to the average baseline value of each slice preparation. All values are reported as mean ± SEM. Statistical comparisons of PPF and LTP were made using the paired t-test. In all cases, *p*<0.05 was considered to be significant. In behavioral experiments, all values are reported as the mean ± SEM. For the analysis of inhibitory avoidance performance, the comparison among behavioral trials within the same group was carried out by using Wilcoxon test. Whereas, the results from locomotor activity studies were analyzed by Student's t test. We considered *p*<0.05 to be statistically significant.

## Results

### Genotyping results

**Figure 1:**
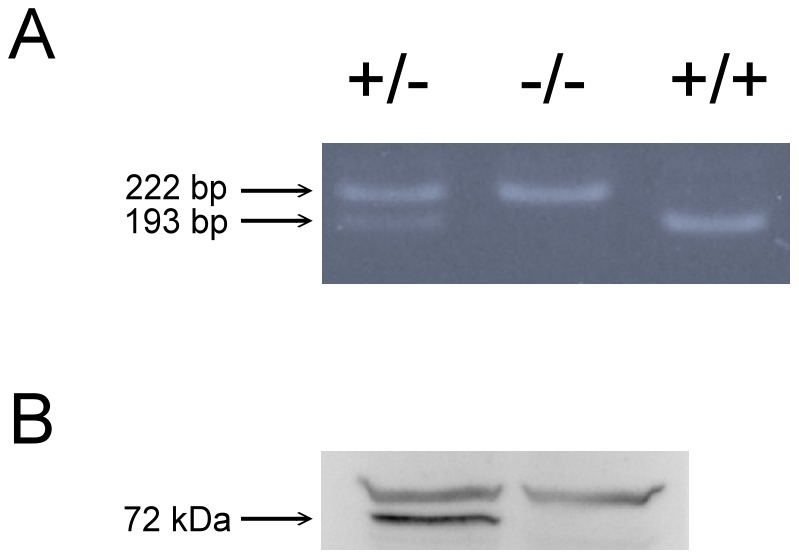
Summary of genotyping results. (A) Representative data obtained from genotyping of wild-type (+/+), heterozygous (+/−) and homozygous (−/−) fishes was validated by polymerase chain reaction. (B) Brain tissues were analyzed by western blot using an FMRP specific antibody. Lane 1 contains wild-type (WT) and Lane 2 contains *fmr1−/−* (KO). The arrow points at FMRP located. The FMRP protein is completely absent in *fmr1−/−*.

Homozygous mutants were obtained with the expected frequency of 25%, and they had normal appearance. The sex ratio in the homozygote population was not significantly different from the other genotypes. The knockout phenotype was confirmed at the DNA level by PCR. The PCR products derived from the wild-type and *fmr1* KO fish were cleaved to 193-and 222-bp DNA fragments respectively (Figure 1A). The protein level was also analyzed by Western blotting, where no FMRP protein was detectable in testes of *fmr1* KO fish (Figure 1B).

### Anxiolytic-like responses in *fmr1* KO zebrafish

**Figure 2:**
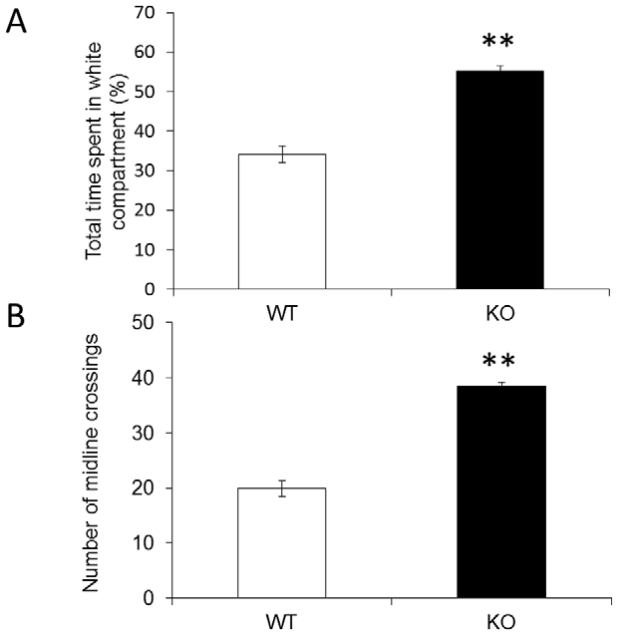
Anxiolytic-like responses of *fmr1* KO zebrafish. (A) Bar graphs of the time spent in the white compartment by *fmr1* KO and wild-type fish. **p<0.01 compared with wild-type fish. (B) Bar graph of the number of midline crossings for *fmr1* KO (n = 12) and wild-type fish (n = 10). **p<0.01 compared with wild-type fish.

The light/dark test has been proposed as a model of anxiety-like behavior in zebrafish. The time spent in white compartment and the numbers of midline crossings were analyzed for each fish. As illustrated in Figure 2, we found a significant genotypic difference in both measures. *fmr1* KO fish spent more time in the white compartment (Fig. 2A, *p*<0.01) and had greater numbers of midline crossings compared to wild-type fish (Fig. 2B, *p*<0.01), indicating lower anxiety and increased locomotion in KO fishes.

### Impaired inhibitory avoidance learning in *fmr1* KO zebrafish

**Figure 3:**
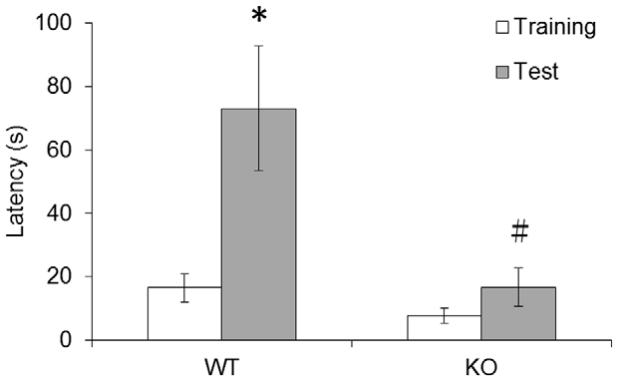
The inhibitory avoidance of *fmr1* KO and wild-type fish. Bars indicate the mean latencies ± the SEMs to cross from the shallow to the deep compartment (in seconds) in the training and test sessions for both genotypes. *p<0.05 compared with training sessions; ^#^ *p*<0.05 compared with wild-type fish.

The inhibitory avoidance test has been extensively used for assessing memories of aversive experiences. In this study, *fmr1* KO and wild-type fish were trained in the inhibitory avoidance learning task, and latency to enter the deep compartment was assessed 24 h after training. As illustrated in Figure 3 the difference between the latencies in the training and test sessions for wild-type was statistically significant (Fig. 3, n = 10, *p*<0.05). In contrast, no significant difference was observed in the *fmr1* KO fishes. Additionally, the retention test was significantly different (*p*<0.05) between wild-type and *fmr1* KO fish.

### Hyperactivity in *fmr1* KO zebrafish

**Figure 4:**
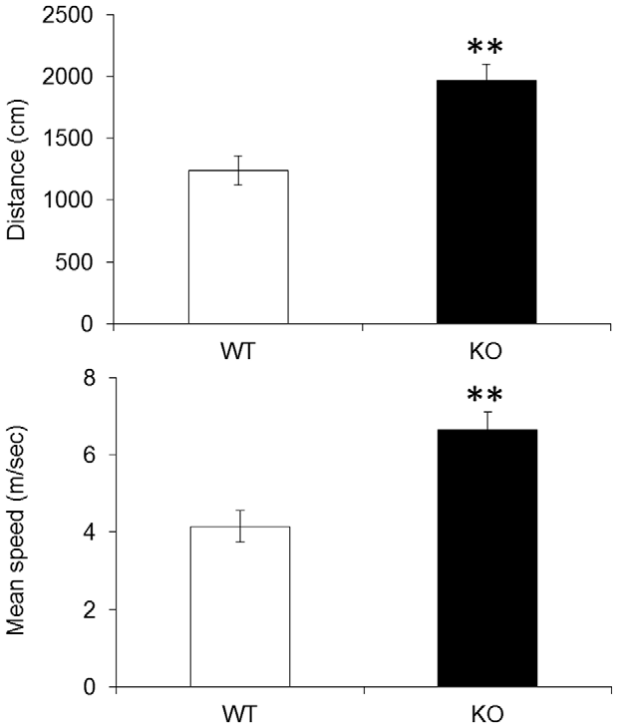
Locomotor activity of *fmr1* KO and wild-type fish. Bar graphs of the total distance moved (in cm) and mean speeds (in m/sec) of *fmr1* KO and wild-type fish. **p<0.001 compared with wild-type fish.

Hyperactivity is the most common symptom of FXS patients and *fmr1* KO mice. To determine whether genotypic differences in locomotor activity were present between genotypes, the total distances swam and mean speeds of *fmr1* KO and wild-type fish were calculated in an open field apparatus for 5 min. As shown in Figure 4, the total distances moved and the mean speeds of *fmr1* KO fish were higher than those of wild-type fish (*p*<0.001 for both outcomes).

### Basal synaptic transmission and PPF in *fmr1* KO zebrafish

**Figure 5:**
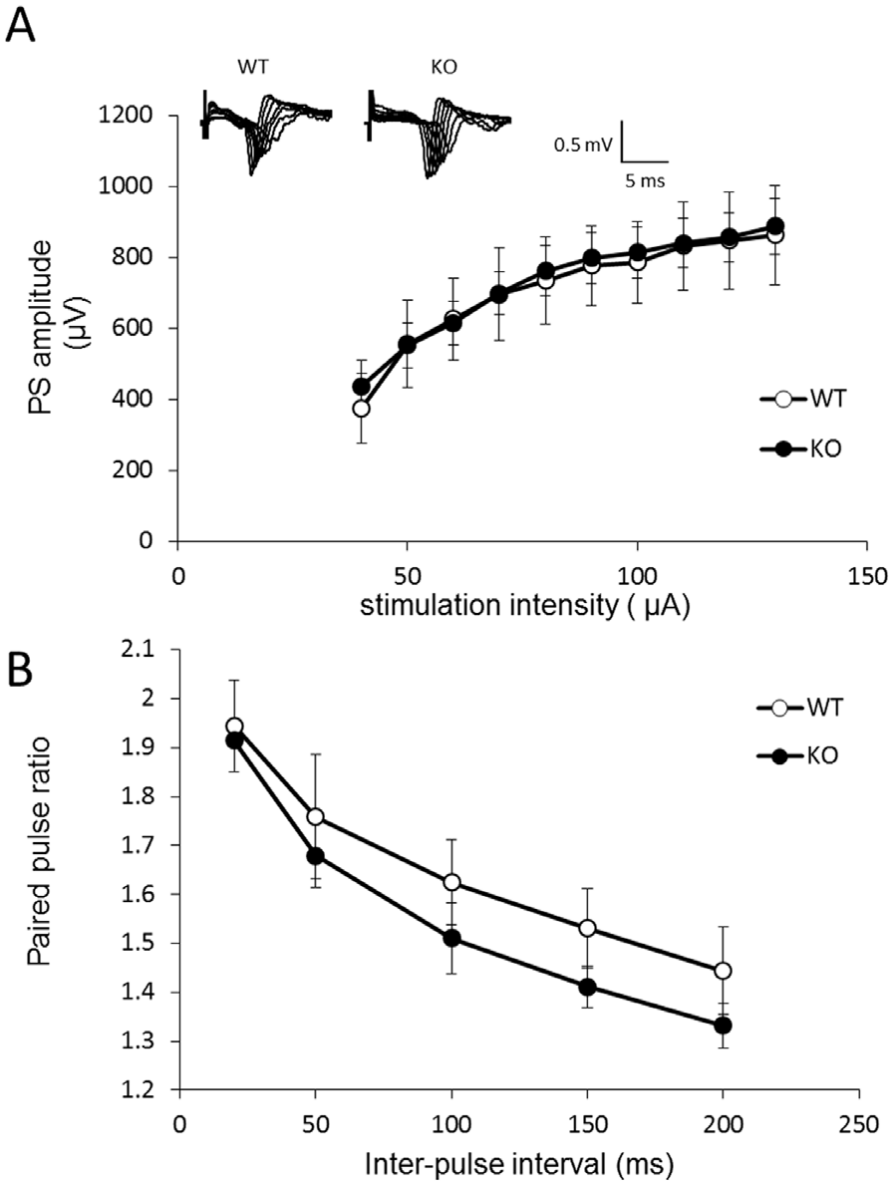
Basal synaptic function is not different between *fmr1* KO and wild-type fish. (A) Summary of the input-output curves that were created by comparing PS amplitude and stimulus intensity (40–130 µA)(n = 6). (B) Paired-pulse facilitation (FFP) was measured by applying paired stimuli and quantifying the facilitation of the second potential relative to the first as a function of the inter-pulse interval (<200 ms)(n = 7).

Basal synaptic transmission at the Dl-Dm synapse was measured by field potential responses to increasing stimulation intensities. As shown in Figure 5A, the amplitude of the population spikes obtained from wild-type and *fmr1* KO slices were compared, and no significant difference between genotypes was noted. Additionally, paired pulse facilitation (FFP) was measured in slices from both genotypes. As shown in Figure 5B, pairs of presynaptic fiber stimulation pulses delivered at inter-pulse intervals of 20, 50, 100, 150 and 200 milliseconds evoked nearly identical amounts of PPF in slices from wild-type and *fmr1* KO zebrafish. We suggest that basal glutamatergic transmission and presynaptic function at the Dl-Dm synapse remain normal in *fmr1* KO zebrafish

### Synaptic plasticity in *fmr1*KO zebrafish

**Figure 6:**
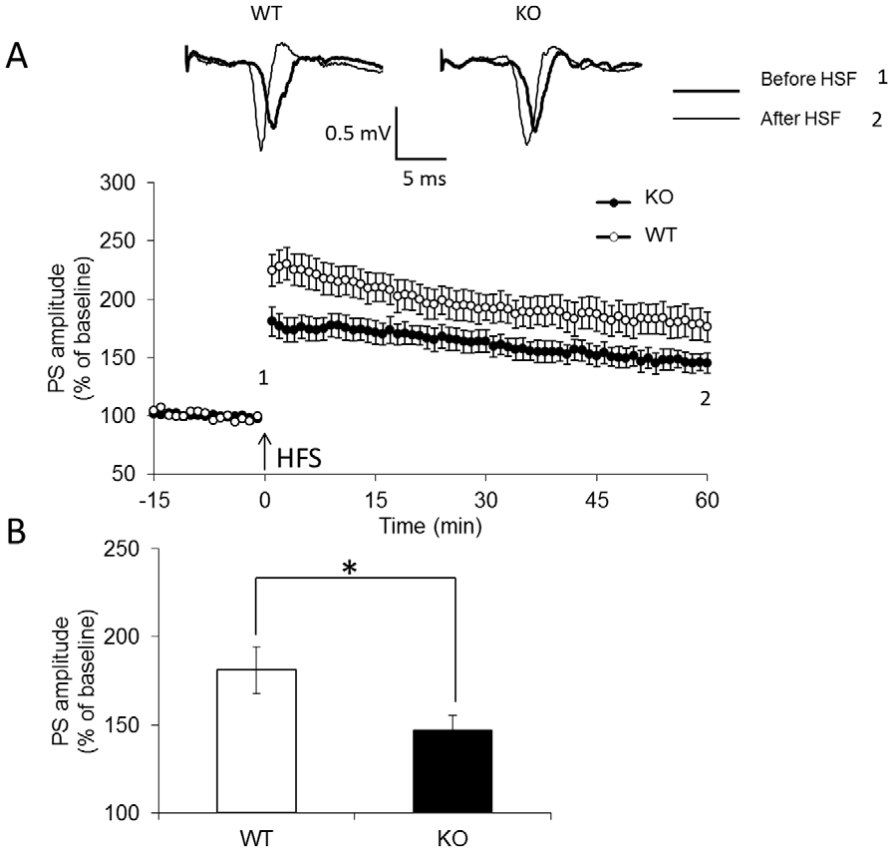
LTP was significantly reduced in *fmr1* KO zebrafish. (A) The arrow indicates delivery of HFS. Insets are representative, superimposed, single sweeps before and after LTP induction in wild-type (n = 9) and *fmr1* KO (n = 10) zebrafish. (B) Summary of the averaged magnitudes of LTP. Bars correspond to the percentages of baseline PS amplitudes during the last 10 min. *p<0.05 compared with wild-type.

**Figure 7:**
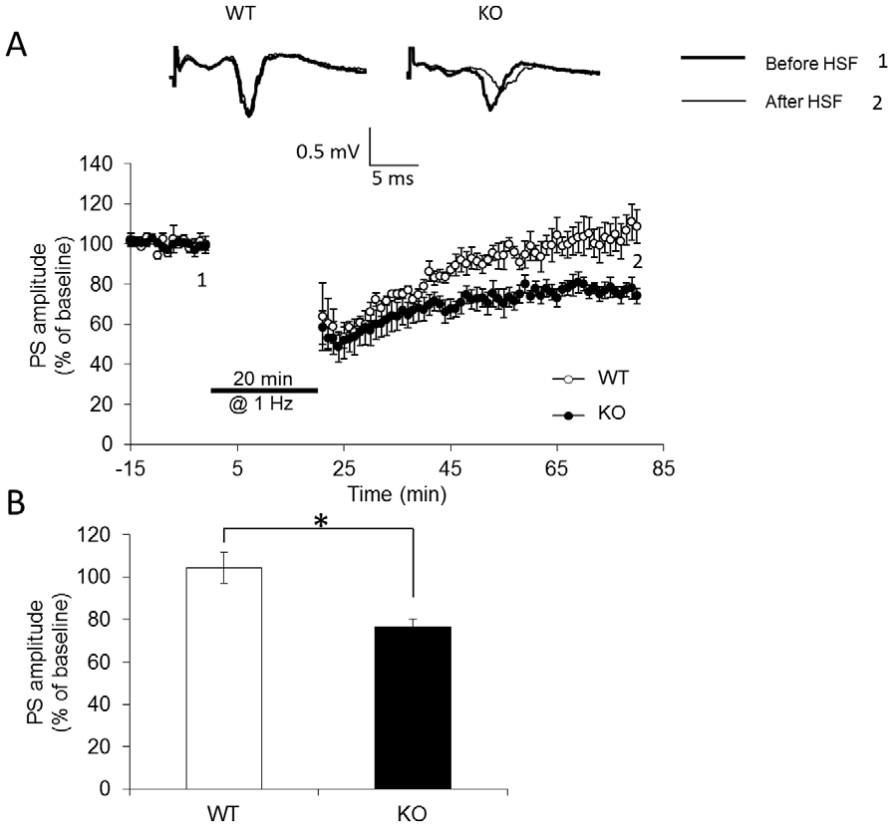
LTD was significantly enhanced in *fmr1* KO zebrafish. (A) LTD was induced by a 20 minute LFS (1 Hz) protocol. Insets are representative, superimposed, single sweeps before and after LTD induction in wild-type (n = 4) and *fmr1* KO (n = 6) zebrafish. (B) Summary of the averaged magnitudes of LTD. Bars correspond to the percentages of baseline PS amplitude during the last 10 min. *p<0.05 compared with wild-type.

In zebrafish, FMRP is highly expressed in the telencephalon [33], an important brain region involved in synaptic plasticity and learning and memory processes. This fact raises an intriguing possibility that FMRP is involved in synaptic plasticity. We next examined whether the loss of FMRP function in zebrafish was related to modulation of synaptic plasticity; to do this, long-term potentiation (LTP) and long-term depression (LTD) were characterized. As shown in Figure 6, LTP was induced by a standard protocol with three trains of high frequency stimulation. LTP magnitude was significantly reduced in *fmr1* KO zebrafish (181.0±7%, n = 9 in wild-type vs. 146.8±6%, n = 10 in *fmr1* KO, *p*<0.05; Fig. 6). LTD is a long-lasting decrease in the synaptic response of the same synapses following prolonged low-frequency stimulation (LFS). LFS-induced LTD was enhanced in slices from *fmr1* KO fish compared to slices from wild-type fish (104.3±7%, n = 4 in wild-type vs. 76.5±5%, n = 6 in *fmr1*KO, *p*<0.05; Fig. 7). These findings suggest that FMRP plays an important functional role in regulating telencephalic synaptic plasticity in zebrafish.

## Discussion

Fragile X syndrome (FXS) is caused by loss of the fragile X mental retardation protein (FMRP). To understand the molecular and cellular pathogenesis of FXS, the disease has been successfully modeled in mice [14], [38], *Drosophila* [37] and zebrafish [33]. In the present study, using *fmr1* KO zebrafish, we were able to investigate the functional role of the *fmr1* gene in mediating cognitive behavior and synaptic plasticity at the Dl-Dm synapse in the telencephalon of zebrafish. Our results can be summarized as follows: (1) *fmr1* KO fish exhibit anxiolytic-like behavior, impaired emotional learning, and hyperactivity, and (2) electrophysiological recordings from telencephalic slice preparations of *fmr1* KO fish showed markedly reduced LTP and enhanced LTD compared with wild-type fish. This study provides the first evidence that FMRP is involved in cognitive functions and telencephalic synaptic plasticity in zebrafish and suggests that zebrafish are a new genetic model system to study Fragile X syndrome (FXS).

Previous behavioral studies have demonstrated that *fmr1* KO mice replicate many of the human behavioral features of FXS, including hyperactivity, learning deficits, impaired social interaction, and abnormal anxiety-related responses [14]. Furthermore, behavioral profiles are a critical first step toward understanding the function of *fmr1*. Here, we performed a series of behavioral analyses on the *fmr1* KO zebrafish that included the light/dark test, the inhibitory avoidance test, and the open-field test to further characterize the consequences of the absence of FMRP.

Interestingly, significant behavioral differences were detected in the light/dark test. Compared with wild-type fish, *fmr1* KO fish had reduced anxiety-related responses in the light/dark test. Our results are remarkably consistent with previous studies [13], [38], [39], [40], [41] in which the loss of FMRP has been reported to be related to anxiolytic responses in mice. Moreover, *fmr1* KO zebrafish show a significantly greater number of crossed lines in the lit compartment, which significantly contributed to locomotor activity. Thus, hyperactivity may be present in *fmr1* KO zebrafish.

Cognitive impairment is a common symptom of FXS patients and FXS mouse models. For instance, Liu et al. (2011) noted impaired inhibitory avoidance acquisition in the *fmr1* KO mice [13]. Here, using an inhibitory avoidance test, we evaluated whether *Fmr1* null mutant zebrafish exhibited learning and memory impairments. Consistent with the notion that FMRP is involved in certain types of learning and memory, we found a significant impairment in the inhibitory avoidance (IA) task in *fmr1* KO fishes. These results suggested that the absence of FMRP might disrupt the detection abilities of and/or the response of the brain's fear system. After the retention test in the IA task, the animals were subjected to an open-field test; activity in the open-field is often used as a measure of exploration in zebrafish. The distance traveled and the mean speed in the open-field task was significantly higher in *fmr1* KO fishes. Our behavioral analyses of the *fmr1* KO fish in the light/dark and open-field tests supports previously reported results [13], [14], [39], suggesting that the absence FMRP expression leads to hyperactivity or increased exploratory behavior.

According to neuroanatomical and behavioral analyses, the telencephalic pallium is a key component of the fear circuitry of teleost fish. For example, goldfish with lesions to the telencephalon have impaired avoidance conditioning [42], [43]. In a previous study, we reported that the physiological function of the telencephalon is involved in the process of fear memory formation in inhibitory avoidance tasks in zebrafish [34]. Furthermore, electrophysiological evidence has demonstrated that the intratelencephalic connections between the lateral and medial pallium, and the Dl-Dm synapse, play important roles in the synaptic plasticity of the zebrafish [36]. Because our behavioral results suggested that the lack of FMRP caused inhibitory avoidance learning deficits, we hypothesized that FMRP may play an important functional role in telencephalic synaptic plasticity.

LTP is an enduring enhancement of synaptic efficacy that has been reported as a cellular model for learning, memory and neuronal plasticity in mammals [44], [45], [46], [47] and zebrafish [36], [48]. Recently, abnormal LTP of excitatory transmission has been found in the hippocampus [49], cortex [50], [51], and amygdala [19] of *fmr1* KO mice. In the present study, we found that the lack of FMRP results in a reduction in LTP at the Dl-Dm synapse, but absence of FMRP did not affect basal transmission function. These findings are in line with previous studies that have described reductions in hippocampal LTP of *fmr1* KO mice [52]; these studies suggest that the absence of FMRP may contribute to reduced hippocampal LTP in *fmr1* KO mice via reduction of NMDAR-mediated currents [53]. In addition, FMRP is required for NMDA receptor-dependent LTP in the mouse prefrontal cortex [19]. Our previous work showed that telencephalic LTP at Dl-Dm synapses is NMDAR-dependent [36]. Therefore, we suggest that the reduction of LTP in the present study may be mediated through an NMDAR-dependent pathway.

In contrast to LTP, long-term depression (LTD) is a long-lasting weakening of synaptic strength that is due to the internalization of AMPA receptors. A recent finding that is of importance for understanding the mechanisms by which FMRP deficiency exerts its effects is the reliable observation of LTD enhancement in the hippocampus of *fmr1* KO mice. For example, a lack of FMRP produced enhanced metabotropic glutamate receptor-dependent LTD [54], [55]. Using low frequency stimulation (LFS) protocol, we found that the lack of FMRP significantly enhanced LTD at Dl-Dm synapses in *fmr1* KO fish compared to wild-type fish. These results may be explained by the mGluR theory of fragile X mental retardation, which suggests that an exaggerated mGluR pathway may lead to an increase in the internalization of AMPAR, as AMPAR trafficking is a driving process for enhanced LTD [56]. The influence of the absence FMRP expression on LTP and LTD leads us to suggest that FMRP may be involved in synaptic function and plasticity.

In summary, we used zebrafish as a system model, taking advantage of available transgenic lines and developmentally very similar to mammals. In addition, it is easy to breed in large numbers and relatively cheap to maintain. Which made it an idea model for prescreen potential therapeutic *in vivo*. We found abnormal behaviors in *fmr1* KO fish that are consistent with findings in humans with defective *fmr1* and *fmr1* KO mice. These findings include anxiolytic-like responses such as increased exploratory behavior in light/dark and open-field tests and avoidance learning impairments. In addition, our recent study has revealed abnormal telencephalon synaptic function (in both LTP and LTD) that may be linked to the behavioral defects displayed in *fmr1* KO fish. Thus, this study suggests that zebrafish is valuable as a potential complementary vertebrate model for the study of the molecular pathogenesis of human fragile X syndrome.
